# Bioengineering Systems for Modulating Notch Signaling in Cardiovascular Development, Disease, and Regeneration

**DOI:** 10.3390/jcdd8100125

**Published:** 2021-09-30

**Authors:** Angello Huerta Gomez, Sanika Joshi, Yong Yang, Johnathan D. Tune, Ming-Tao Zhao, Huaxiao Yang

**Affiliations:** 1 Department of Biomedical Engineering, University of North Texas, Denton, TX 76207, USA; AngelloHuertaGomez@my.unt.edu (A.H.G.); SanikaJoshi@my.unt.edu (S.J.); Yong.Yang@unt.edu (Y.Y.); 2 Texas Academy of Mathematics and Science, University of North Texas, Denton, TX 76201, USA; 3 Department of Physiology & Anatomy, University of North Texas Health Science Center, Fort Worth, TX 76107, USA; johnathan.tune@unthsc.edu; 4 Center for Cardiovascular Research, Abigail Wexner Research Institute, Nationwide Children’s Hospital, Columbus, OH 43215, USA; Mingtao.Zhao@NationwideChildrens.org; 5 The Heart Center, Nationwide Children’s Hospital, Columbus, OH 43215, USA; 6 Department of Pediatrics, The Ohio State University College of Medicine, Columbus, OH 43210, USA

**Keywords:** Notch signaling, cardiovascular cells, bioengineering, microfluidics, hydrogel, spheroid, 3D bioprinting, organoid, single-cell omics

## Abstract

The Notch intercellular signaling pathways play significant roles in cardiovascular development, disease, and regeneration through modulating cardiovascular cell specification, proliferation, differentiation, and morphogenesis. The dysregulation of Notch signaling leads to malfunction and maldevelopment of the cardiovascular system. Currently, most findings on Notch signaling rely on animal models and a few clinical studies, which significantly bottleneck the understanding of Notch signaling-associated human cardiovascular development and disease. Recent advances in the bioengineering systems and human pluripotent stem cell-derived cardiovascular cells pave the way to decipher the role of Notch signaling in cardiovascular-related cells (endothelial cells, cardiomyocytes, smooth muscle cells, fibroblasts, and immune cells), and intercellular crosstalk in the physiological, pathological, and regenerative context of the complex human cardiovascular system. In this review, we first summarize the significant roles of Notch signaling in individual cardiac cell types. We then cover the bioengineering systems of microfluidics, hydrogel, spheroid, and 3D bioprinting, which are currently being used for modeling and studying Notch signaling in the cardiovascular system. At last, we provide insights into ancillary supports of bioengineering systems, varied types of cardiovascular cells, and advanced characterization approaches in further refining Notch signaling in cardiovascular development, disease, and regeneration.

## 1. Introduction

Notch signaling is a pathway that stems from an evolutionary trait that regulates the fate of the cell in differentiation, proliferation, immune response, and angiogenesis. Initiation of the pathway starts with the interaction between the Delta-like ligand (Dll) 1, 3-4, Jagged ligand (Jag) 1 and 2 from one cell and Notch receptors (Notch1–4) from the other cell [1] The interaction triggers the cleavage of the Notch intracellular domain (NICD), which then relocates to the nucleus for further transcriptional regulation and cell fate determination. 

To serve its role in the cardiovascular system, the Notch signaling pathway has the task of coordinating intercellular events between groups of neighboring cells [2]. How these cells communicate with the binding of Notch-associated receptors and Jag/Dll associated ligands is highly dependent upon the characteristics of the cell type in question and the cell type’s ability to send or receive certain signals. The Notch transmembrane receptor transits into the cytoplasm following activation, and into the nucleus, then, it acts as a transcription factor [3]. This cellular distribution allows for many potential interactions between signaling and binding cells. In order to understand how the Notch pathway regulates cardiovascular development, disease, as well as regeneration and repair after injury, the contribution of individual cell types must be considered [4]. These cell types involved in the Notch signaling pathway include endothelial cells (ECs) [5], smooth muscle cells (SMCs) [6], fibroblasts (FBs) [7], cardiomyocytes (CMs [8]), immune cells [9], and hiPSC-derived cardiovascular cells [10]. 

The formation of the cardiovascular system is controlled by Notch signaling as well as the formation of blood vessels. Notch signaling activation is necessary for trabecular development, atrioventricular canal boundary formation, and cardiac neural crest cell development [11]. Targeting the Notch pathway can also be seen as a new therapeutic approach to treating many congenital heart diseases (CHDs). Mutations in human Notch signaling genes have been found to be involved in several diseases with cardiac involvement including bicuspid aortic valve disease [12], left-sided CHD [13,14], and Adams–Oliver syndrome. Notch2 and Notch3 are also associated with disease—Alagille syndrome and leukoencephalopathy, respectively [15].

The majority of findings in Notch signaling as summarized above are based on findings from animal models and a few clinical studies. Accordingly, understanding of the mechanisms of Notch signaling in the human cardiovascular system is still inconclusive, and progress is limited by the lack of ideal in vitro human models. To date, the advances of bioengineering systems and cell recourses from human-induced pluripotent stem cells (hiPSCs) provide unprecedented opportunities for precisely modeling and modulating Notch signaling in in vitro human cardiovascular systems, such as defined shear stress-induced mechanotransduction and crosstalk between co-cultured human cardiovascular cells in a microfluidics system [16], bioactive hydrogel for stem cell delivery in the injured myocardium [17], 3D spheroid culture [18], exosome and cell secretome for cardiovascular regeneration [19], [20], and 3D bioprinting of biomaterials and cardiovascular cells [21]. 

This review focuses on the current understanding of the Notch signaling pathway in the cardiovascular system as well as advanced bioengineering systems for better modulating the Notch signaling in human in vitro cardiovascular systems. First, specific Notch signaling pathways involved within each type of cardiovascular cell are summarized. Secondly, the current engineering systems, including microfluidics, hydrogel, 3D bioprinting, exosome and cell secretome, and spheroid, applied in controlling Notch signaling in the varied cardiovascular cells, are discussed. Finally, the perspectives on applying most advent bioengineering systems and characterization approaches in the single-cell and tissue levels on cardiovascular development, disease, and regeneration are further emphasized.

## 2. Notch Signaling in Cardiovascular Cells

### 2.1. Endothelial Cells

ECs are an essential part of the cardiovascular system by serving as constituents of blood vessels where they regulate homeostasis via blood fluidity and fibrinolysis, vascular tone, angiogenesis, and platelet aggregation [5]. Notch signaling is able to modulate the existing state as well as the development of the cardiovascular system by first impacting the biological functions of ECs [22]. One example is how the interaction between Notch and the vascular endothelial growth factor (VEGF) signaling pathway plays a key role in angiogenesis [23]. The VEGF pathway induces Notch activity such that the Notch signaling pathway can limit angiogenesis by reducing the number of ECs sprouting from parent vessels [24]. In the mature heart chamber, the endocardium, a specialized endothelial tissue that is continuous with the vascular endothelium and lines the interior of the heart, forms the cardiac valves and the coronary vessel endothelium [25]. Additionally, crosstalk between miRNA and the Notch1 pathway also shows an important role in regulating endothelial cell angiogenic activity [26]. In a mice myocardial infarction (MI) model, it was demonstrated that silencing of the miR-34 family suppresses Notch1, resulting in weakened MI-induced pathological left ventricular remodeling and improved cardiac function with increased angiogenesis [27]. The early endocardium expresses many Notch elements which interact with the myocardium for proper cardiac development. Notch inactivation in the endocardium via the targeted mutants in *RBPJk* or *Notch1* results in decreased expression of the ligand Dll4, leading to the malformation of the ventricular chamber [28]. In addition, Notch signaling is essential to vascular EC stabilization and EC heterogeneity establishment, as can be seen in the Notch pathway’s role in preventing ECs from undergoing apoptosis. This is apparent in rat cardiac allograft vessels where impaired *Notch4* expression leads to endothelial dysfunction during the development of transplant arteriosclerosis (TA) [29]. In brief, the Notch pathway is integral to ensuring endothelial function and vascular development in the heart. 

### 2.2. Smooth Muscle Cells

SMCs, another important cardiovascular cell type associated with the Notch signaling pathways, are responsible for arterial contraction and extracellular matrix (ECM) production [6]. In the tunica media of the vasculature, SMCs have an important part in the elastic rebound of an artery in response to a hemodynamic condition [30]. For Notch signaling to occur, heterotopic interactions between endothelial signal-sending cells and smooth muscle receiving cells must take place [31]. As such, to regulate the development and function of cardiac vasculature, the Notch signaling would coordinate EC and SMC behaviors. Notch signaling can control, specifically, vascular SMC development in various ways. For example, Notch signaling upregulates the differentiation of cardiac neural crest cells to SMCs. Therefore, when Notch4 receptor activity in these neural crests is inhibited, the results involve the defective formation of the smooth muscle layer of aortic arteries. leading to heart defects such as aortic arch patterning defects and pulmonary artery stenosis [32]. On the other hand, the Notch intracellular domain (NICD) of Notch1, Notch2, and Notch4, by expressing downstream gene target HEY, can use a negative feedback loop to inhibit vascular SMC differentiation [33]. There is also evidence of transcription regulation of miRNAs in the phenotype of VSMC. Induction of miR-143/145 through Jag-1/Notch signaling in human VSMC is in need of Notch-induced acquisition of the contractile phenotype, suggesting that Notch signaling also controls gene expressions indirectly associated with VSMC maturation [34]. Further studies that use genetic inactivation strategies in mice show that Notch signaling is essential to the vessel wall’s maturation along the arterial tree [35]. This information aligns with how mutations in Notch receptors can lead to several SMC-related vascular diseases. For example, dominant mutations in the Notch3 receptor cause cerebral autosomal dominant arteriopathy with subcortical infarcts and leukoencephalopathy (CADASIL) and small vessel disease (SVD), which are characterized by progressive loss of SMCs [36]. Additionally, Alagille syndrome, an autosomal dominant multisystem disorder, is another disorder caused by a mutation in the *JAG1* gene. According to the work by Hofmann and coworkers, *Jag1* in mice, conditional deletion of *Jag1* in SM22α-expressing cells of the developing portal vein mesenchyme was sufficient to recapitulate the hepatic defects of Alagille syndrome [37]. Furthermore, Notch signaling also has a role in EC–SMC crosstalk. For vascular smooth muscle cells (VSMCs) to mature completely, the endothelial expression of Jag1 is essential in ECs through EC–VSMC crosstalk via Notch3–Jag1 receptor–ligand binding [38,39]. Changes in Notch1 endothelial activation also play an important part in managing VSMCs phenotypic switching in vascular injury [40]. In summary, the contribution of Notch signaling in SMCs to vascular development and disease is growing in pathophysiological importance.

### 2.3. Cardiac Fibroblasts

Cardiac fibroblasts (CFs) in a healthy heart maintain the balance between the synthesis and degradation of collagen and other ECM components [7]. In wound healing after myocardial injury, CFs are triggered to start differentiating into myofibroblasts [41]. Notch1 signaling has an inhibitory effect on these CFs, which are essential for the maintenance of proper myocardial function as well as response to injury. For instance, Notch has an important role in cardiac fibrosis as well as in other organ systems. Notch gene induction can initiate fibrosis by activating alpha-smooth muscle actin (α-SMA) transcription as well as myofibroblast transformation [42]. Thus, when Notch signaling is inhibited by DAPT, a-secretase inhibitor, profibrotic factor expression is decreased, and fibrosis can be prevented or reverted [43]. Another example of the Notch1 pathway’s effect on CFs comes from how the pathway is impacted by the overexpression of calreticulin (CRT), a protein proven to be involved in the pathogenesis of various cardiovascular disease (CVD) and development. The overexpression of CRT is correlated with the activation of Notch signaling in human CFs, making CRT a novel target to treat Notch-associated cardiac fibrosis [44]. Additionally, Notch signaling can connect CFs and other cardiovascular cells. In fact, in transgenic mice overexpressing the *Jag1* ligand on the surface of CMs, the Notch pathway is activated in adjacent CFs. The study demonstrates how Notch activation in CFs may be targeted to restrict both cardiac hypertrophy and fibrosis [45]. Notch3 also interacts with other signaling pathways such as the RhoA/ROCK signaling pathway to regulate CF activity by connecting CFs and ECs: it activates the RhoA/ROCK pathway in endothelial barrier dysfunction [46]. In conclusion, Notch signaling in CFs not only regulates cardiac fibrosis upon heart injury, but also fuels cell-to-cell interactions between cardiovascular cells, such as SMCs, CMs, and ECs.

### 2.4. Cardiomyocytes

CMs are responsible for generating contractile force and working with other cardiac cells (ECs, CFs, etc.) to regulate the rhythmic beating of the heart [8]. Notch signaling is responsible for inducing CM proliferation and can even increase CM population size. Notch1 signaling inhibition in Notch1-deficient cardiovascular cells can both promote or inhibit CM differentiation. Even though Notch1 is not expressed in CMs, the effects of NOTCH1 on cardiac differentiation and proliferation may be derived from the intercellular communication between cardiomyocytes and neighboring noncardiac cells [4]. This shows that technologies for Notch signaling can be useful for generating differentiated cardiomyocytes in a clinical setting [47]. In rat neonatal CMs, high proliferation correlates with high *Notch* expression and low proliferation correlates with negligible *Notch* expression [48]. Moreover, the Notch1 intracellular domain is acetylated in proliferating neonatal rat CMs. By affecting Notch1’s half-life and transcriptional activity, this acetylation controls both the amplitude and duration of Notch1 signaling in CMs. As a result, the reversible acetylation of Notch1 has been identified as a target to regulate the proliferative potential of CMs [49]. The manipulation of Notch1 expression levels through the target of miR322 via the FBXW7 gene also has potential therapeutic application for cardioprotection against ischemia which causes a loss of cardiomyocytes [50]. Additionally, Notch1 signaling can control the electrical behavior of cardiomyocytes through the actions of the NICD [51]. This allows Notch to remodel the action potentials in adult CMs while keeping neonatal CMs in an immature electrophysiological state, thus effectively regulating myocardial mechanical behavior. Furthermore, the role of Notch signaling in CMs extends to cardiac regeneration. In zebrafish, following the amputation of the ventricular apex, three notch receptors in the endocardium and epicardium begin expression. When Notch signaling, in general, is inhibited in the regenerative window, there is a significant negative impact on scar formation and myocardial regeneration at the amputation site. This is because Notch inhibition causes a significant decrease in CM proliferation necessary for regeneration. Surprisingly, hyperactivation of the Notch pathway leads to a similar decrease in CMs, confirming the sensitivity of zebrafish cardiac regeneration to changes in Notch signaling [47]. From CM differentiation to regeneration, Notch is essential for the proper activity in this cell type. 

### 2.5. Immune Cells 

Although communication between the heart and the immune system is not fully understood, immune cells are certainly highly integrated through crosstalk with cardiovascular cells via specific cardiokines, cytokines, hormones, and neurotransmitters [9]. Notch signaling has an important role in leading both immune cell development and function in the heart. Notch1 aids in determining cell-lineage commitment in development lymphocytes; Dll4 drives thymic T cell commitment after interaction with Notch1 [52]. Further revealing the significance of Notch1 in alloimmunity, a study testing the effect of a protein Notch1 blockade in an MHC-mismatch mouse cardiac transplant model finds that anti-Notch1 treatment prolonged allograft survival [53]. This suggests that Notch1 can be potentially used to develop new methods for immunosuppression for heart transplantation. Additionally, the ligand Dll4 can induce T-cell activation and proliferation through interaction with the Notch1 receptor present on Naive CD4^+^ T cells [54]. Since most human plaques are CD4 T-helper cells, this means that Notch receptors 1 through 4 influence the cardiovascular system by regulating immune responses during the stages of atherosclerosis, a chronic autoimmune inflammatory disease [55]. Notch signaling also plays a profound role in the onset of atherosclerosis by modulating macrophage polarization into M1 (high inflammatory) or M2 (anti-inflammatory) subtypes. Notch1 induces an increase in mitochondrial oxidation, which triggers the expression of M1 proinflammatory genes [56]. Notch1 signaling in monocytes, as well as vascular macrophages, causes inflammation by not only facilitating this proinflammatory M1 phenotype but also doing so at the expense of the anti-inflammatory M2 phenotype and inhibiting M2 differentiation. In summary, the interactions between Dll4 and Notch1 in T-cells and macrophages are key mediators in cardiovascular inflammation and also warrant future investigations.

### 2.6. hiPSC-Derived Cardiovascular Cells 

**Figure 1:**
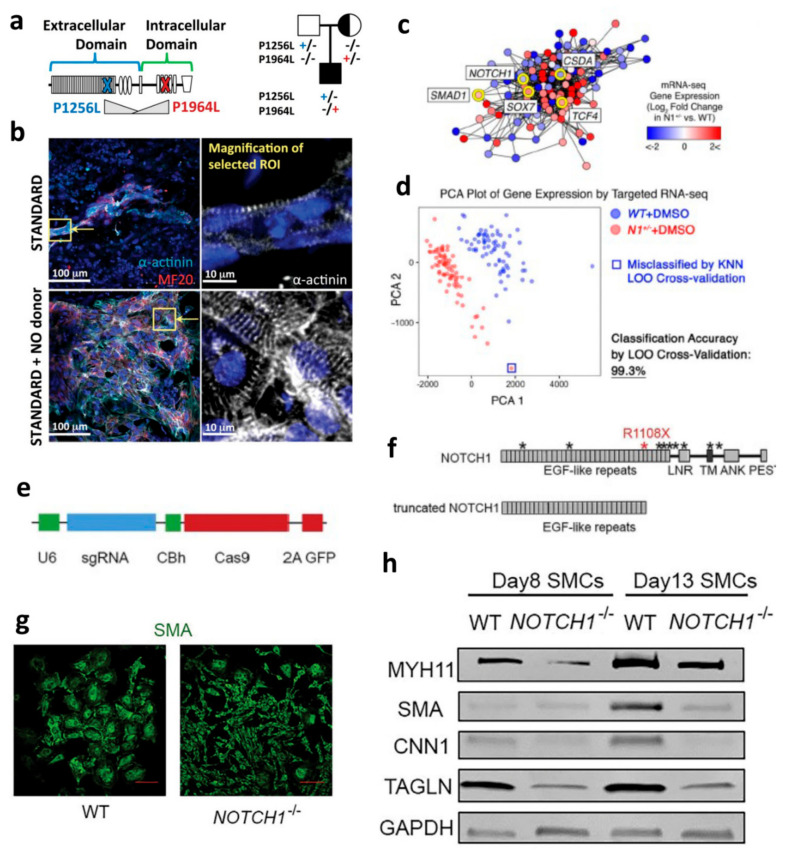
Notch signaling in varied cardiovascular cells for modeling cardiovascular development and disease. hiPSC-CMs: (**a**) HLHS patient-derived iPSCs show Notch1 variants carried by the mother, father, and proband [58] (**b**) α-actinin immunofluorescent staining of HLHS patient-derived hiPSC-CMs with NO donor NONOate have an increase in cardiomyocyte yield [58]. Reproduced with permission from [Sybil C. L. Hrstka et al.,], [Stem Cells]; published by [John Wiley and Sons], (2017). **hiPSC-ECs:** (**c**) Map of the gene network dysregulated by N1 haploinsufficiency in hiPSC-derived ECs [60]. (**d**) Principal component analysis (PCA) plot of gene expression in *WT* (*n* = 72) or *N1^+/−^* (*n* = 79) ECs [60]. Reproduced with permission from [Christina V. Theodoris et al.,], [Science]; published by [The American Association for the Advancement of Science], (2021). **hiPSC-SMCs:** (**e**) Structure of CRISPR/Cas9 and sgRNA plasmid [64]. (**f**) NOTCH1 protein structure and truncated NOTCH1 after targeting by CRISPR/Cas9 and sgRNA [64] (**g**) Impaired human SMC differentiation due to Notch1 knockout [64] (**h**) Western blot of expressions of *MYH11*, *SMA*, *CNN1*, *TAGLN*, and *GAPDH* in SMCs differentiated from NCSCs with WT and *NOTCH1*^−/−^ [64] Reproduced with permission from [Jiao Jiao et al.,], [The Journal of Thoracic and Cardiovascular Surgery]; published by [Elsevier], (2018).

Human-induced pluripotent stem cells (hiPSCs) have recently attracted more attention and applications in cardiovascular development, disease modeling, and regenerative medicine [10]. hiPSCs are also found as an ideal in vitro human model for studying the role of Notch signaling and Notch variants in cardiovascular pathogenesis [57]. For example, hiPSCs derived from a hypoplastic left heart syndrome (HLHS) patient with Notch1 mutations in the extracellular and intracellular domains exhibit Notch1-dependent nitric oxide (NO) signaling deficiency and impaired cardiogenesis [58]. Each parent contributed a distinct Notch1 mutation, but the proband had both (Figure 1a). The researchers also found that NO donor (NONOate) supplementation in Notch mutant iPSCs could alter cardiomyocyte yield (Figure 1b). This was used to conclude that there is an NO-dependent signaling component in the Notch1 pathway needed for cardiovascular cell lineage specification. Additionally, extending the applications of in vitro modeling of Notch with hiPSCs to a clinical setting may lead to the identification of a therapeutic target for patients with functionally compromised Notch variants. For example, Notch1, Notch2, and Notch3 can be activated by adding Jag1 peptide ligands during the differentiation of HLHS patient-derived hiPSC, restoring both cardiomyocyte differentiation capacity and SMC formation [59]. Calcific aortic valve disease (CAVD), the third most common form of CVD, is also linked to Notch1 mutations and as a result, the similar approach of targeting the normalization of core Notch1 pathway components can be used for therapeutic applications [60]. Similar to the aforementioned HLHS-affected hiPSCs, the architecture of dysregulated networks in disease-relevant cells differentiated from patient-derived hiPSCs were used to screen for small molecules which correct the abnormal gene network responsible for CAVD (Figure 1c). Replicates of isogenic *WT* and N1-haploinsufficient ECs treated with DMSO or small molecules allowed the researchers to map the gene network regulated by N1 more confidently than in experiments with fewer observations of the network state (Figure 1d). Theodoris et al. leveraged hiPSCs and machine learning technologies to identify therapeutic candidate XCT790, which was able to limit valve thickening by correcting the network dysregulation in genetically defined iPSC-derived ECs [60]. Furthermore, hiPSCs have also opened the opportunity to investigate the genetic mechanisms of Notch-related cardiac diseases such as CHD by using patient-specific cardiac cells, including all the cell types outlined previously. For instance, syndromic CHD such as Alagille syndrome is caused by a single-gene defect (a variation in the Notch2 gene) [61]. Currently, hiPSC models are increasingly being used to study the contribution of genetic variation in Alagille syndrome development [62]. In the future, genome editing techniques may be used to correct variants in hiPSCs so that the same can be done in isogenic disease-affected cardiac cells [63]. In bicuspid aortic valve patients, Jiao et al. used CRISPR/Cas9 genome-edited hiPSCs to evaluate the role of a Notch1 mutation in SMCs (Figure 1e,f). In hiPSC-SMCs in which Notch1 was targeted and truncated, SMC differentiation was impaired (Figure 1g). The gene expressions of contractile proteins (MYH11, SMA, CNN1, and TAGLN) were lower in these truncated Notch1 groups as well (Figure 1h). Overall, hiPSC-derived cardiovascular cells have brought up unprecedented opportunities to help us better understand Notch-associated CVDs, especially children and adult CHDs, and potential treatments. 

## 3. Bioengineering Systems for Modeling Notch Signaling in the Cardiovascular System

Bioengineering systems are able to apply the fundamental engineering principles in biological science and medicine [65]. Bioengineering systems have become increasingly useful in the research and translation of cardiovascular development, disease modeling, and regenerative medicine. Various bioengineering systems, such as microfluidics, hydrogels, spheroids, and 3D bioprinting have been used. Bioengineering systems guide researchers in understanding human development and diseases by mimicking human physiological responses, thereby replacing animal studies and creating solutions to human diseases [66]. Herein, the focus of this section is to summarize and discuss the current bioengineering systems used in developing cardiovascular models for deciphering the roles of Notch signaling pathways in cardiovascular development, disease, and regeneration.

### 3.1. Exosomes and Cell Secretome

Exosomes are biological vesicles (ranging from 50 to 100nm) responsible for transporting proteins, lipids, and nucleotides to targeted cells [19]. The targeted cell receives the exosome and modification of its phenotype initiates by the presence of microRNAs (miRNAs) in exosome content [67]. This method of intercellular communication is an alternative to direct cell-to-cell interaction. Recent studies reveal the role of exosomes secreted from stem cells to have a reparative state on injured or damaged tissues via Notch signaling. One study demonstrated the effectiveness of using exosomes derived from hiPSCs with miRNA-199b-5p to induce angiogenesis and increase blood perfusion in mice with ischemic limbs with miRNA inhibiting Jag1–Notch1 signaling [68]. A similar study found the activation of adipose-derived MSCs (ADMSCs) by serum exosomes in response to myocardial infarction (MI) in mice [69]. The exosomes in MI mouse displayed upregulation of miR-1956, thereby increasing VEGF signaling for targeting the downregulation of Notch1 gene and protein in MSCs to promote angiogenesis [69]. Exosomes offer another important route for providing intercellular communication via Notch signaling to promote regeneration on damaged tissues.

Similarly, cell secretomes from the conditioned medium of cell culture refer to the secretion of paracrine factors from cells that include cytokines, chemokines, and growth factors [20]. The paracrine factors allow communication to cells and can limit the communication to specific cells [20]. Cell-conditioned media contains the collection of paracrine factors secreted by specific cells that could be used as a therapeutic agent [70]. A study by Ribeiro da Silva et al. cocultured neonatal mice CMs with a CFs-conditioned medium to understand the influence of CFs secretomes on CM differentiating into the ventricular conduction system cells (conduction phenotype). The results indicate that fibroblast secretomes are necessary for the activation of Notch1 to induce the conduction phenotype of the CMs [71]. Moreover, the promotion of valve formation through Notch activation is found to be modulated by the endocardial secretome. The endocardial secretome via activation of Dll4–Notch1 promotes epithelial-mesenchymal transition (EMT) and cell migration in the heart valve [72].

### 3.2. Microfluidics System

**Figure 2:**
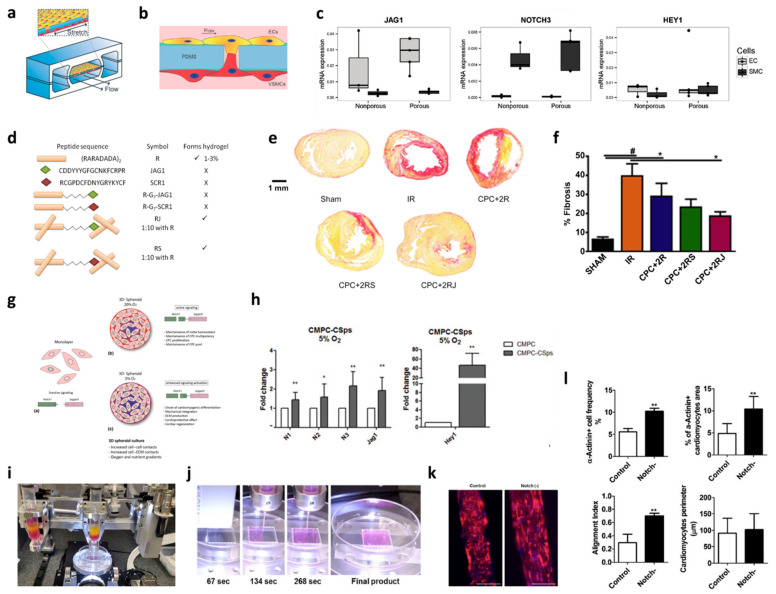
Modeling Notch signaling in various bioengineering systems. Microfluidics system: (**a**) ECs-VSMCs physiological microfluidic design; (**b**) ECs-VSMCs interaction via pores; (**c**) expression of Notch3, Jag1, and Hey1 in ECs and VSMCs [76]. Reproduced with permission from [Nicole C. A. van Engeland et al.,], [Lab Chip]; published by [Royal Society of Chemistry], (2018). **Hydrogel**: (**d**) Composition of hydrogels with and without Jag1; (**e**,**f**) Comparison of fibrosis formation with various hydrogels, * *p* < 0.05, ** *p* < 0.01, # *p* < 0.001 [82]. Reproduced with permission from [Archana V. Boopathy et al.,], [Biomaterials]; published by [Elsevier], (2014). **Spheroid**: (**g**) Representation of Notch signaling in monolayer and 3D spheroid conditions; (**h**) expression of Notch1, Notch2, Notch3, Jag1, and Hey1 between monolayer culturing (CMPC) and 3D spheroid culturing (CMPC-CSps under hypoxia conditions), * *p* < 0.05, ** *p* < 0.01 [84]. Reproduced with permission from [Arianna Mauretti et al.,], [MRS Communications]; published by [Springer Nature], (2017). **3D bioprinting:** (**i**) Custom 3D bioprinter containing three components for cardiac construct; (**j**) interval image sequence of cardiac tissue construct formation; (**k**) comparison of bioprinted tissue construct with and without Notch inhibition; (**l**) Measurement of α-actinin positive cells, cardiomyocyte area, alignment of cardiomyocytes, and cardiomyocyte perimeter among control and Notch inhibition tissue constructs for cardiac tissue development, ** *p* < 0.05 compared with control [91]. Reproduced with permission from [Zhan Wang et al.], [Acta Biomaterialia]; published by [Elsevier], (2018).

Microfluidics allows for the controlling of fluids that are confined to micro-sized dimensions [16]. Manipulation of the microenvironment with the microfluidics systems provides spatial and temporal positioning, precise nutrient/cytokine/drug supply to cells, and dynamic force cell culture [73]. Tiemeijer et al. used microfluidics to regulate vascularization of ECs by microcontact patterning Dll4. Specifically, the removable microfluidics device was designed for allowing seeding the ECs in a specific pattern relative to the distribution of Dll4 ligands. The effectiveness of Dll4 influence on EC-promoted vascular expansion through the Notch–Dll4 receptor–ligand bindings was validated in this microfluidics system [74]. Sharghi-Namini et al. explored angiogenesis formation via microfluidics, which enabled the controlled observation of interactions between Dll4-exosomes and ECs sprouting [75]. The microfluidic device consisted of two channels (one for cell seeding and the other for culture media) and sandwiched a channel composed of a collagen matrix with posts on each side of the channel interface. A VEGF gradient was created over the collagen matrix, allowing the seeded cells to sprout. Control of Dll4-exosomes concentration demonstrates the diffusion through the collagen to the ECs sprouting site, inhibiting the growth of the ECs [75]. Another factor associated with microenvironment manipulation is fluid flow shear stress (FSS). A human-lung-on-a-chip microfluidic device was engineered to mimic the blood vessel physiology through ECs and vascular SMCs (VSMCs) interactions (EC-VSMC) and signaling via FSS as shown in Figure 2a [76]. Upon hemodynamic loading, the EC-VSMC microfluidics device mimics vascular cell behavior and the stress and strain that blood vessels experience under forces. Hemodynamic forces were found to be necessary for the differentiation of ECs–VSMCs interactions into the vessel wall formation when the shear force induces EC to VSMC signaling via Jag1–Notch3, as shown in Figure 2b,c. This platform presents the further evaluation of the device for physiological and pathological studies on vascular walls by the FSS and Notch signaling relationships [76]. Further work in FSS demonstrates the inclusion of microfluidics to create a microphysiological environment of the aorta for drug screening metformin [77]. The microfluidic device consists of one gas channel and one media channel. A thin polydimethylsiloxane (PDMS) membrane between the two channels is seeded with human pluripotent stem cell-derived aortic smooth muscle cells (hPSC-ASMCs), and the gas channel enables the mechanics of the aorta. The results reveal a decrease in aortic aneurysm progression due to the presence of the Notch1 protein. Furthermore, the Notch1 receptor functions as a biomechanical sensor in blood vessels and suggests a correlation between Notch1 and biomechanical forces (shear stress) from blood flow [77]. The present works using the microfluidic system display how Dll4 distribution influences ECs growth and vascularization, and how Notch1 signaling influences the VSMCs fate by EC-VSMC interaction and shear stress-induced mechanosensitivity. The current work describes the role of blood flow in triggering Notch signaling, indicating Notch as a mechanosensor [76,78]. Microfluidics is an excellent bioengineering system that is reproducible and cost-effective for understanding the Notch signaling involved in the physiology and pathology of vascular networks. 

### 3.3. Hydrogels

Hydrogels are one type of most significant biomaterials recapitulating the in vivo microenvironment for 3D cell culturing, benefits for cell proliferation, differentiation, migration, nutrient transport, oxygen permeability, and spatial environment that resembles the extracellular matrix [79,80,81]. Gerbin et al. fabricated an injectable hydrogel with Dll1 used to promote CM proliferation after transplantation through activation of Notch1 signaling [17]. The hydrogel is composed of collagen type I crosslinked with anti-IgG for Dll1 immobilization that leads to Notch1 activation in human embryonic stem cell-derived cardiac progenitor cells (hESC-derived CPCs), thereby influencing cell fate in hESC-derived CPCs. Collagen I-Dll1 hydrogel shows increased CM proliferation, increased graft size, and reduces the decline of systolic function of acute myocardial infarction (MI) on rats in comparison to those in the collagen-only group [17]. Moreover, a comparison between immobilized and non-immobilized ligands showed a decrease in Dll1-Notch1 activation, whereas immobilized ligands had increased Dll1–Notch1 activation, emphasizing the importance of immobilizing Dll1 on the collagen hydrogel [17]. Similarly, Boopathy et al. evaluate the mouse ESC-derived CPCs seeded in a self-assembled peptide (SAP) hydrogel as a therapeutic agent on a rat MI model [82]. The peptide of the SAP hydrogel mimics Jag1, as shown in Figure 2d. It is found that Notch1 activation is dependent on hydrogel stiffness, as the property influences cardiogenic expression in mouse ESC-derived CPCs and reducing fibrosis, as shown in Figure 2e,f. The effects of using the SAP hydrogel without cells in a myocardial infarction rat model are demonstrated to improve cardiac function with decreased fibrosis [83]. In summary, hydrogels offer an additional platform for the 3D culture of cardiovascular cells for exploring the possibilities in regeneration on myocardial infarction via the activations of Notch1–Dll1 and Notch1–Jag1 receptor–ligand bindings in the CMs and CPCs.

### 3.4. Spheroids 

Spheroids are another 3D cell culture technique of creating an in vivo-like microenvironment by providing multicellular cell–cell and cell–ECM interactions [18]. The Notch signaling pathways of Notch1, Notch2, Notch3, Jag1, and Hey1 were studied in the spheroid culture of human CPCs, which include both adult and fetal human CPCs from adult and fetal human hearts, respectively, under normoxia and hypoxia conditions [84]. Under normoxia, the fetal CPCs spheroids showed upregulation of Notch2, Notch3, Jag1, and Hey 1 compared with a 2D monolayer culture, whereas adult CPCs spheroids displayed upregulation of Notch1, Notch3, and Hey 1. The findings indicate an increase in Notch signaling activation in the 3D spheroid culture due to the in vivo-like microenvironment (Figure 2g). Under hypoxia conditions, fetal CPCs spheroids showed an increased expression in all Notch components and Hey1 (Figure 2h), whereas adult CPCs spheroids displayed upregulation in Notch1, Notch3, and Hey 1 when compared to the respective monolayer culture [84]. Enhancement of Notch signaling is achieved under hypoxia conditions due to the 3D environment and cell-ECM interactions. The cells in spheroids are tightly compact and allow for the secretion of ECM proteins that can influence cell behavior in the microenvironment [84,85]. Moreover, the CPC spheroids were transplanted to recapture Notch1-Jag1 signaling or repair right ventricular heart failure in an athymic rat model. The aggregated cells improved right ventricular function of the rats due to the increase in Notch1 signaling, responsible for the CPC differentiation into a reparative state. Furthermore, exosomes from the spheroid reduced fibrotic gene expression, including CTGF, VIM, and COLIA1 in rat cardiac fibroblasts, as well as increased tube length in human umbilical vein endothelial cells (HUVECs) in comparison to those of both the monolayer culture and exosome-free spheroids [86]. Spheroids provide direct interactions between cells and cell and ECM, replicating the physiological environment of a tissue to a conventional 2D culture. The current work of using spheroids in activation of Notch signaling demonstrates a regenerative state of myocardial infarction with the CPCs treatment.

### 3.5. Bioprinting

Bioprinting is a promising tool for manufacturing complex 3D tissue constructs to mimic in vivo tissue architectures and composition and harnessing the manipulation of the microenvironment to understand tissue development and function [21]. The extrusion-based method is commonly used in bioprinting due to superior print speed [87]. Bioprinting ushers’ advantages, including patient-derived anatomical shape via computed tomography (CT) images, forming complex organization structure of desired tissue, manipulating cell distribution in a printed construct (Figure 2I,j) [88,89,90]. Furthermore, the effect of Notch signaling inhibition on a bioprinted cardiac patch was studied. Application of DAPT on the cardiac patch displayed synchronized contraction due to inhibition of Notch signaling. The inhibition stimulated the maturation of CMs and decreased the proliferation state of neonatal CMs (Figure 2k,l) [91] Progression in bioprinting has provided a feasible and flexible platform to create multicellular constructs and allow intercellular crosstalk via Notch signaling in a defined 3D cell/tissue culture. Although the current work of bioprinting with Notch signaling modulation displays a pivotal role in cardiac regeneration and maturation, further research is necessarily needed for applying Notch signaling on cardiovascular regeneration and treatment.

## 4. Perspectives

The molecular and cellular mechanisms of Notch signaling in the regulation of human cardiovascular development, disease modeling, and regeneration are still inconclusive. The purpose of this review paper is to emphasize the advantages of using bioengineering systems for modulating and modeling Notch signaling pathways for cardiovascular research better and more precisely. Although the summarized works of establishing the microfluidics, hydrogel, spheroid, and 3D bioprinting to realize a range of Notch receptor and Jag/Dll ligand bindings among the human cardiovascular cells are preliminarily explored for cardiovascular development, disease modeling, and regenerative medicine, broader and better applications of the advanced bioengineering systems with various types of cardiovascular cells (CMs, SMCs, ECs, CFs, immune cells, and hiPSC derivatives) are extensively recommended. For example, it is well-known that Notch3 mutations are responsible for CADASIL, however, there is a lack of knowledge in the mechanisms of CADASIL contributed with the cardiovascular cells [92]. For example, hiPSC-derived SMCs and ECs from CADASIL patients could be co-cultured in a microfluidics system to model Notch signaling dysregulations in CADASIL [76]. Three-dimensional bioprinting and spheroid provide the construction of complex structures to mimic the human heart at the tissue level. For example, the vascularized engineered heart tissue with ECs, SMCs, and CMs would be an ideal model for studying Notch signaling in varied pathophysiological situations, such as molecular interferences of Notch inhibitors and activators, induced fluid flow, and passive or active mechanical stretching [78,93,94,95,96]. Moreover, Notch mutation and signaling are closely associated with the maldevelopment of heart and CHDs, such as HLHS and left ventricular noncompaction (LVNC). A human heart in vitro developmental model, like hiPSC-derived cardiac organoids, would fill the gap for disclosing the molecular and cellular mechanism of Notch signaling-modulated cardiovascular development and CHDs. Cardiac organoids directly derived from hiPSCs self-organize into a heterogeneous 3D construct, recapitulating the multicellular and spatiotemporal development of the human heart in vitro [4,97,98]. Employing an hiPSC-derived organoid system in modeling the Notch signaling of cardiovascular maldevelopment potentially provides additional insights and discoveries of new targeted pathways for CHD treatments.

The bioengineering systems also show promising potential for clinical translation on Notch signaling-associated treatments of cardiovascular diseases. The microfluidics systems were applied to explore the mechanism involved with decreasing the progress of aortic aneurysm via drug screening metformin. This mechanism of Notch1 signaling activating the cell contractile phenotype discovered in microfluidics can be used to support the use of metformin for treating aortic aneurysms in patients [77]. Hydrogels with the Jag1 ligand in treating MI could be also potentially translated into clinical studies due to the effectiveness and small invasive procedure of the injectable hydrogel [83]. The CPC spheroids have further shown the possibility to translate clinically for the treatment of heart failure with the faster fabrication of spheroids and minimal configurations. CPCs have already demonstrated safety and cardiac function improvement in clinical trials for treating ventricular dysfunction [99]. The end goal of bioprinting is the clinical application of a patient-specific tissue construct for repair and replacement. Although application is far away, progress is achieved, as in the work of Wang et al. creating an accelerated matured cardiac tissue construct through Notch inhibition. This progress can increase the likelihood of clinical trials with further research in their work [91]. 

To better understand the biology of Notch signaling modeling in the cardiovascular bioengineering system, advanced characterization approaches, such as single-cell omics and 3D culture imaging and preparation (tissue clearing and light-sheet fluorescence microscopy) [100,101] would be useful to dissect the unclear or unknown mechanisms of Notch signaling pathways in multicellular crosstalk and 3D cardiovascular structure. Collectively, the potential combinations of bioengineering systems could open new avenues of deeper and better understandings of Notch signaling in human cardiovascular development, disease, and regenerative.

## 5. Conclusions

**Figure 3:**
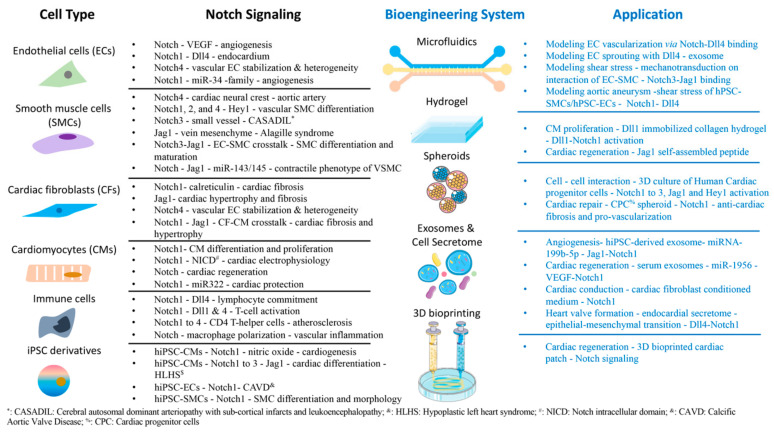
The pivotal roles of Notch signaling in varied cardiovascular cells with bioengineering systems.

Notch signaling plays a critical role in cardiovascular development, disease modeling, and regeneration. Nearly all cardiovascular-related cell types are regulated by Notch signaling directly or indirectly. Due to the limitations of animal models and clinical studies, bioengineering systems using various human cardiovascular cells have gradually been established for modulating Notch signaling pathways specifically for cardiovascular research, as summarized in Figure 3, such as a microphysiological blood vessel with Notch3–Jag1 interactions between ECs–VSMCs, a microphysiological aorta with Notch1 activation, angiogenesis influence by Notch1–Dll4 interaction, heart regeneration assisted by hydrogels, exosome and conditioned culture medium, and 3D spheroids with Notch1–Dll1 and Notch1–Jag1 activations in CMs and CPCs, and maturation of CMs in 3D bioprinted tissue construct by Notch inhibition. Overall, bioengineering systems offer microenvironment modulation for archiving unique and unprecedented opportunities of delineating the molecular and cellular mechanism of Notch signaling in cardiac development and disease and providing targeted therapies for cardiovascular regeneration.

## Data Availability

Not applicable.
